# Robotic‐assisted hip and knee revision arthroplasty: A scoping review

**DOI:** 10.1002/jeo2.70285

**Published:** 2025-05-26

**Authors:** Michele Venosa, Giandomenico Logroscino, Emilio Romanini, Gianpiero Cazzato, Giuseppe Petralia, Andrea Vespasiani, Giacomo Placella, Patrizio Caldora

**Affiliations:** ^1^ RomaPro Center for Hip and Knee Arthroplasty, Polo Sanitario San Feliciano Rome Italy; ^2^ GLOBE, Italian Working Group on Evidence Based Orthopaedics Rome Italy; ^3^ Department of Life, Health and Environmental Sciences University of L'Aquila L'Aquila Italy; ^4^ Department of Mini‐Invasive and Computer‐Assisting Orthopaedic Surgery San Salvatore Hospital L'Aquila Italy; ^5^ Department of Orthopaedics and Traumatology San Raffaele Hospital, Università Vita‐Salute San Raffaele Milan Italy; ^6^ Orthopaedic Department San Giuseppe Hospital Arezzo Italy

**Keywords:** hip, joint replacement, knee, revision arthroplasty, robotic‐assisted arthroplasty

## Abstract

**Purpose:**

Technological advances have significantly revolutionised orthopaedic surgery over the past decades. The introduction of robotic‐assisted (RA) systems in total joint arthroplasty (TJA) surgery, especially in total hip arthroplasty (THA) and total knee arthroplasty (TKA), represents a key innovation. While the advantages of robotic assistance in primary joint replacement surgery are relatively well known, its application in hip and knee revision surgery implies a more complex and challenging scenario. The procedures needed are inherently more difficult compared to primary arthroplasties because of considerable bone loss, scar tissue, compromised anatomical landmarks, and at times even damaged or eroded joint structures.

**Methods:**

This scoping review synthesises existing literature on the application of RA systems in revision hip and knee arthroplasty. A systematic search on the six major databases in September 2024 identified 24 eligible studies for inclusion.

**Results:**

Although various studies and case reports have demonstrated the successful use of robotics in TJA surgeries, the existing body of literature concerning revision surgery is still limited, and many questions remain unanswered. While, for instance, robotic systems seem to have held some promise for better improvement in implant positioning and alignment, it is still quite unclear whether this development in technological advancement will translate into better long‐term outcomes such as improved implant longevity and lower revision rates.

**Conclusions:**

Although early data are promising, having some possible short‐term advantages, wide diffusion is limited because of high costs, significant training requirements, and limited long‐term outcome data. By identifying gaps in the current literature and emphasising areas for future investigation, this review aims to define the ongoing development and refinement of RA applications in revision arthroplasty, ultimately seeking to determine whether these technologies can achieve sustainable improvements in implant longevity and patient satisfaction.

**Level of Evidence:**

Level III.

**Abbreviations**
BCS: Bicruciate‐stabilised
CT: computed tomography
KOOS: Knee Injury and Osteoarthritis Outcome Score
PRISMA: Preferred Reporting Items for Systematic Reviews and Meta‐Analyses
RA: robotic‐assisted
RA‐TKA: robotic‐assisted total knee arthroplasy
RTKA: revision total knee arthroplasty
THA: total hip arthroplasty
TJA: total joint arthroplasty
TKA: total knee arthroplasty
UKA: unicompartmental knee arthroplasty

## INTRODUCTION

The last few decades have seen significant advances in surgical technology for orthopaedic surgery [5, 8, 36]. Presumably, the application of robotic‐assisted (RA) systems in total joint arthroplasty (TJA), especially in total hip arthroplasty (THA) and total knee arthroplasty (TKA), has been one of the most important evolutions in this medical field [18, 21, 31, 42, 49]. These technologies have been developed to overcome the limitations of conventional manual surgical procedures, thus potentially improving patient outcomes [30, 40, 41]. The main advantage of the robotic systems is their ability to integrate preoperative imaging and/or intraoperative data recording with intraoperative execution to achieve more effective restoration of joint biomechanics [15]. In primary TJA, robotic systems such as MAKO®, ROSA®, ROBODOC®, NAVIO® and CORI® can improve surgical accuracy, reduce the variability of implant alignment and human error, as well as provide optimisation of component positioning [2, 7, 12, 14, 29, 32, 48]. These improvements result in superior short‐term clinical outcomes such as lower complication rates, improved functional recovery, and increased patient satisfaction. Whereas the potential benefit of robotic assistance in primary arthroplasty has been very well documented [22, 35, 55], the application in revision surgeries—particularly in hip and knee revisions—is much more complex and presents a challenging landscape.

Revision arthroplasty is often required due to the failure of primary implants because of loosening, instability, infection, and other complications [9, 45, 51]. Such procedures are intrinsically more challenging compared to primary arthroplasties as there is potential bone loss, scar tissue, compromised anatomical landmarks and sometimes damaged and eroded joint structures. Moreover, stable fixation and appropriate alignment of the implants in revision surgery represent the keystones of the procedure, as improper positioning may result in a series of further catastrophic complications, such as instability, reduced mobility, and the failure of the implant [25, 46, 47, 52]. Conventional revision techniques, though effective for all intents and purposes, often lack the precision required to deal with such multi‐faceted problems [16, 44].

Hence, this is where RA surgery has started to show considerable potential. Over the last years, robotic systems have been adapted for use in revision TJA, and early evidence suggests that these technologies can offer substantial advantages. By providing more accurate preoperative planning due to enhanced imaging, robotic systems support the development of customised surgical plans that address the unique anatomical challenges presented by each patient. Intraoperatively, robotic systems help surgeons execute these plans with remarkable precision limiting human error, minimising the need for intraoperative adjustments, and preserving as much bone stock as possible [4, 20, 38, 39, 58]. These features are important in revision surgery since maintaining the structural integrity of the remaining bone is critical in ensuring long‐term implant success.

Despite such promising developments, the use of RA systems in hip and knee revision surgery is still in its infancy. Although there are case reports and retrospective studies concerning the application of robotics in arthroplasty revision surgery, the literature is still limited, and many questions are unanswered. For instance, while robotic systems have proven to improve implant positioning and alignment, it is not clear whether such improvements will translate into better functioning long‐term results, such as longevity of the implant and low revision rates.

This scoping review aims to outline the current status of RA revision arthroplasty for hip and knee joints. Based on a critical analysis of the available literature, this study will discuss the benefits and shortcomings of robotic systems in performing revision surgeries, compare these outcomes to conventional techniques, and then identify gaps in the existing research.

## MATERIALS AND METHODS

This scoping review was conducted in accordance with the Preferred Reporting Items for Systematic Reviews and Meta‐Analyses (PRISMA) framework. It aims to synthesise the available literature on the use of RA systems for revision hip and knee arthroplasty. The methodology encompasses an extensive search with a selection of studies, including data extraction, and an analytical process that is detailed below.

### Search strategy

A literature search was systematically carried out across six major databases—PubMed, Embase, the Cochrane Library, Google Scholar, ResearchGate and Semantic Scholar—from their inception to September 2024. The search strategy included Medical Subject Headings terms and keywords related to the subject, such as ‘robotic‐assisted arthroplasty’, ‘revision total joint arthroplasty’, ‘revision hip arthroplasty’ and ‘revision knee arthroplasty’. There were no limitations regarding languages, although the search was limited to studies only involving human subjects. Moreover, reference lists of all the studies included were manually screened to identify other relevant publications that might have been missed in the main searches.

### Eligibility criteria

**Figure 1:**
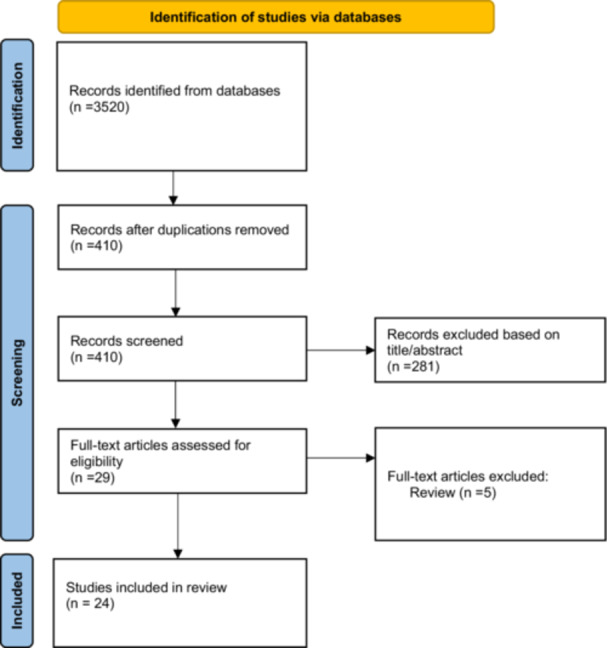
PRISMA workflow. PRISMA, Preferred Reporting Items for Systematic Reviews and Meta‐Analyses.

Studies were eligible for inclusion if they met the following criteria: (1) involved adult patients undergoing revision TJA using RA technology, either for hip or knee arthroplasty; (2) included any type of study design (randomised controlled trials, observational studies, case reports, etc.) and (3) evaluated the outcomes or techniques of RA revision surgeries. Conference abstracts and full‐text publications were both included, while animal studies or studies not related to RA revision TJA were excluded. At the end of the search strategy and study selection, 24 papers were eligible and were included in this review (Figure 1).

### Data synthesis

A narrative synthesis approach was used to summarise the findings, given the heterogeneity of the study designs and outcomes across the included studies. The focus was on identifying the key themes related to the application of RA systems in revision hip and knee arthroplasty, including surgical technique, surgical precision, clinical outcomes and challenges encountered.

## RESULTS

### Conversion from hip fusion to THA

Adil et al. in 2021 presented a unique case of Stryker MAKO® RA conversion of a surgical hip arthrodesis (performed after two unsuccessful hip surgeries for a femoral neck fracture) to THA [1]. A thorough preoperative planning via thin‐cut computed tomography (CT) scans of the pelvis and hip, a computerised 3D model, and a physical 1:2 scale model permitted them to correctly understand the spatial representation of the hip anatomy before the surgical procedure. The association of pre‐operative data collection and real‐time navigation enabled the acquisition of all necessary data points. After removing the ectopic bone, the side plate, and the hip compression screw, registration of the hip fusion mass was performed. After reaching the appropriate depth of the acetabulum, data points from its medial portion were obtained. With the robotic assistance, a Stryker Tritanium® revision acetabular component was impacted. Different from the preoperative plan, a monolithic Smith & Nephew Redapt™ stem was impacted down the femur to bypass cortical stress risers resulting from the removal of the osteosynthesis system. A dual mobility system (oxynium‐polyethylene) was used to enable a more stable implant. The entire preoperative leg length inequality was not fully addressed to prevent the risk of fractures when testing the intraoperative range of motion. The authors reported a progressive clinical improvement of the patient at the early follow‐ups, even in the presence of a 3‐cm leg length inequality and a residual Trendelenburg gait pattern.

### Conversion from femoral osteosynthesis to THA

Dretakis et al. in 2024 presented the case of a 69‐year‐old female patient suffering a cut‐out complication of a proximal intramedullary nail for a left peri‐trochanteric hip fracture that happened 4 months before [13]. Since the lag screw was prominent in the hip, the surgeons opted for conversion into a THA in a RA procedure (Stryker MAKO®). The robotic procedure was performed according to the same pre‐operative and intraoperative steps of a primary robotic image‐guided procedure. The satisfactory recovery of the patient confirmed the aptness of choice since the robotic revision surgery provided accurate acetabulum component placement and the precise restoration of the hip joint anatomy and biomechanics.

### Cement removal technique in hip arthroplasty revision

Yamamura et al. reviewed the medical records and radiographs of 19 patients (17 women and 2 men) needing the revision of their primary THA (for aseptic loosening, septic loosening, and central migration of the bipolar head) for whom bone cement of the femoral canal was removed using the ROBODOC® system (an active robotic system) [56]. Bone cement was completely removed in all patients, with no instances of perforation or femoral fracture. Full weight bearing was possible within 1 week after surgery in 9 patients and all remaining cases within 2 months.

### Revision from primary THA to revision THA

Bargar et al. described the use of RA THA revision surgery with ROBODOC® [3] for the first time in 1998. In this clinical trial, 30 THA revisions were included, where either cement or fibrous tissue was removed by robotic assistance. The regular Orthodoc™ and the revision programme were used. Both programmes were safe and practical. The regular programme was used in cementless cases or in cemented revisions where only a small cement mantle had to be removed (in 23 patients). After the manual removal of the primary implant, the robot removed bone cement or, in cementless cases, fibrous tissue during the reaming and instantly created the cavity for a new cementless implant. In seven patients, the new revision programme, as a prototype, was used to remove extended cement mantles from the cavity. In this programme, the bone‐to‐cement interface was identified stepwise by the surgeon during the planning for the Orthodoc™ system. After cement removal, a new cemented stem was implanted. The authors concluded that RA surgery could be performed without exposing the patient to uncertain risks.

Zhang et al. in 2022 reported a case study describing the adoption of Stryker MAKO® for the RA revision of a THA associated with a severe acetabular defect (Paprosky Type IIIB) [59]. The preoperative CT planning was useful in generating a patient‐specific 3D printed model of the pelvis to simulate the acetabular component implantation and guide the surgical procedure. The acetabular reaming was performed with the assistance of the robotic arm, allowing the preservation of the remaining acetabular bone mass. A 62‐mm diameter Tritanium® acetabular cup (Stryker) was implanted according to the plan. The bone defect was filled, and the augment was implanted and fixed with three screws according to the 3D‐printed template. The post‐operative quality of life greatly improved with significant recovery of the function, as confirmed by the results of the Harris Hip score and the Western Ontario and McMaster Universities Osteoarthritis Index at 6 months.

In the same way, Zhou et al. in 2021 reported their satisfying experience of 71 patients who had RA hip revision (cup revision carried out in 68 patients and stem revision in 68 patients) [60].

### Revision from medial or lateral unicompartmental knee arthroplasty (UKA) to TKA

Kalavrytinos et al. in 2020 reported the first case of a revision surgery to TKA with the use of the robotic MAKO® system in an 87‐year‐old female affected by the failure of a medial UKA [19]. The bony segmentation was performed as it was a primary TKA, assuming that the femoral and tibial components were the native femoral and tibial profiles. According to the preoperative CT, the estimated depth of the medial plateau was found to be 10 mm more distal than the lateral one. However, during the intraoperative planning, the tibial resection cut was moved 2 mm distally to reach the healthy intact part of the bone and fill the gap of the medial condyle with a 10 mm augment. The authors reported a highly satisfactory success rate for the patient at 1‐year follow‐up.

Wallace et al. in 2020 presented four cases of medial UKA requiring an RTKA (robotically performed with Stryker MAKO®) [54]. The authors reported good post‐operative results with no need for the use of augments thanks to the extreme accuracy of the robotic system.

Yun et al. retrospectively reviewed 34 patients affected by the failure of UKA and who had undergone revision surgery with conventional or RA procedures (with MAKO®) [57]. The authors reported that the mean thickness of the poly in the conventional group was 12 mm while it was 10 mm in the robotic cohort (no statistical difference). Surgery times were comparable in the two groups. No revision components were used in either cohort, and all conversions were executed by implanting primary implants. Augments use showed a statistically significant difference between the two cohorts (29% in the conventional group versus 0% in the robotic one).

Mancino et al. in their prospective study published in 2024 compared 16 patients undergoing RA revision of UKA to TKA and 35 matched patients undergoing RA‐TKA (with MAKO®) [33]. The study reported that robotic arm‐assisted revision of UKA to TKA was a safe procedure. There were no conversions to conventional manual instrumentation during the surgeries, indicating the reliability of the robotic system. Intraoperative blood loss, post‐operative rehabilitation outcomes, limb alignment, length of hospital stay and post‐operative complications were similar in both groups at short‐term follow‐up. RA stereotactic boundaries limited bone resection to the confines of the preoperative surgical plan, with none of the patients requiring additional augments or bone grafts.

Lachance et al. performed a retrospective review on 50 patients undergoing conversion from UKA to TKA, divided into four groups based on primary and conversion surgery: manual‐to‐manual, manual‐to‐robot, robot‐to‐manual and robot‐to‐robot [23]. Manual UKA utilised the Zimmer Biomet Oxford® Partial Knee System, and manual TKA utilised the Zimmer Biomet Persona® System. The Stryker MAKO® was used to perform the robotic procedures (Stryker Restoris® MCK and Triathlon® prostheses were used for UKA and TKA, respectively). Patients who had a primary RA UKA had significantly fewer augments. Notably, robotic revisions had the largest increase in poly size, unlike other studies. This discrepancy, which was not entirely clear, in the authors' opinion, could be related to the intrinsic characteristics of the implants themselves, in converting from a Zimmer UKA to Stryker RA‐TKA. No statistically significant differences between primary RA or manual UKA and RA or manual TKA were detected in terms of range of motion at 1 year, complications, or differences in components.

Tuecking et al. reported a retrospective case‐control study comparing 20 patients undergoing TKA and 20 undergoing revision arthroplasty from UKA to TKA with an imageless RA system (NAVIO®, Smith & Nephew) [53]. The implants (Journey® II BCS prostheses) were functionally aligned with the pre‐arthritic anatomy and preoperative soft tissue balance, with no need for metal augment (femoral augments, stems) in any of the cases. The results of this study showed comparable surgery time, alignment accuracy, bone preservation, and outcomes.

Raja et al. in 2024 reported for the first time the technique and the results of the conversion of UKA to TKA in three patients (for aseptic loosening, stress fracture, and progressive osteoarthritis) employing the ROSA® Knee System (Zimmer Biomet) with a non‐CT based system [37]. The authors reported positive results at 6 months in terms of radiographic stability, pain and function.

### Revision from patellofemoral UKA to TKA

Lachance et al. reported three cases of RA conversion of previous patellofemoral arthroplasty to TKA using the Stryker MAKO® system (for the progression of the osteoarthritis in the other compartments, with no concern for infection or mobilisation of the components) [24]. A preoperative CT scan (according to Stryker Makoplasty Protocol) was obtained in all patients. A metal artefact reduction system assisted in visualising the articular femoral surface during the scan. Intraoperative registration was performed before removing the patellofemoral component, linking the loaded CT data with the array placement to the local anatomy. Standard components (with no augments or revision components) were used for all patients. No intraoperative complications have been mentioned. Good mechanical and early clinical outcomes (as confirmed by KOOS Jr scores) have been reported in all patients.

### Revision from primary TKA to revision TKA (RTKA)

Steelman et al. in 2021 reported the first case of an RA revision with Stryker MAKO® of a TKA due to failure (aseptic loosening of the tibial component) of a primary implant [50]. A Stryker Triathlon® TS arthroplasty with the use of both femoral and tibial cones as well as medial and lateral posterior femoral augments was implanted. The authors reported a satisfactory post‐operative component alignment with excellent clinical results 6 months after the revision surgery.

Danoff et al. described the surgical technique of a 1.5‐stage exchange TKA for periprosthetic joint infection robotically assisted with Stryker MAKO® (based on their experience with six patients) [11]. Intraoperative bone landmark registration was performed with the primary femoral and tibial components in situ. After the removal of the components, the robotic saw was used to perform all bone cuts necessary for the new implant and to debride any bone in contact with prior infection. If a bone defect was identified, the robotic technology was able to simulate a new cut edge at the location of a healthy bone site. The authors reported good early outcomes in this series, considering this approach a promising option with an excellent ability to clear the infection and restore knee function.

Cochrane et al. reported the procedural details concerning RTKA implant using an imageless robotic system (CORI®, Smith & Nephew) [10]. Revisions were performed both for aseptic and septic failures (115 patients). Image‐free registration and surface mapping were performed with the primary insert and components in place. The bone cuts were performed using the robotic system, and the augments were implanted based on the identified bony defects. The results of this cohort (90‐day follow‐up) showed a favourable outcome with few readmissions.

Ngim et al. in 2023 reported a retrospective review of 19 patients who had MAKO® RA‐TKA [34]. The inclusion criteria were: patients who had previously undergone UKA or TKA, or patients with a knee cement spacer with well‐controlled periprosthetic infection. In all cases, the RA revision was performed by implanting a fully cemented TKA implant from the Triathlon® knee revision system (Stryker Orthopaedics). In the CT preoperative planning (performed according to Mako robotic protocol), metal artefact reduction software was used to reduce the artefacts from primary implants. Intraoperative registrations of the femoral and tibial bony landmarks were performed with the existing primary implants in situ. After ligament balancing, the prosthetic components were removed with the MAKO® saw. The bone cuts necessary for the revision implant were the same as those in a primary case. Femoral or tibial augments were implanted based on bony defects. All patients progressively achieved independent ambulation. None of them had uneventful recoveries and did not need further revision surgery.

MacAskill et al. in 2024 reported the results of a series of 25 consecutive patients undergoing an RA RTKA (Stryker MAKO®) [27]. All patients had undergone a conventional non‐robotic TKA (different prosthetic implants had been implanted). The reasons for the revision surgery were: instability, aseptic loosening, and infection. A pre‐operative CT evaluation was performed according to Mako System Protocol, and several attempts have been performed to ensure an overall registration error of ≤0.5 mm due to metal artefacts from the primary implants. On the surgical side, after careful implant removal, the virtual gaps were balanced within the planning software, and bone cuts were performed with the robotic arm. Implant augmentation was used depending on the requirements for gap balancing. At the end of the RA revision surgery, the final position of the RTKA was assessed in terms of femoral component varus/valgus, flexion/extension, internal/external rotation, transepicondylar axis, posterior condylar axis, tibial slope, varus/valgus and internal/external rotation. The coronal, sagittal and axial positions of the primary TKA and those of the RTKA were compared. The results of this study showed a statistically significant difference between posterior condylar offset and tibial slope component positioning for RTKA compared to primary TKA. The post‐operative posterior condylar and trans‐epicondylar axes averaged 0.24° internal rotation and 1.45° external rotation, respectively. These values were slightly less than the preoperative average values of 1.26° external rotation and 2.47° external rotation, respectively. These are statistically significant aspects for a stable RTKA. Other potential benefits of the robotic revision procedure may include appropriate implant sizing, which is fundamental for the resultant ligamentous tension of functional RTKA.

Main findings of the papers included in the present review are displayed in Table 1.

**Table 1:** Summary of the main findings of the studies examined in this review.

Authors	Number of patients	Type of surgery	Robotic system	Main findings of the study
Adil et al. [1]	1	Conversion from hip fusion to THA	Stryker MAKO®	The association of pre‐operative data collection and real‐time navigation enabled the acquisition of all necessary data points to perform the conversion from hip fusion to THA.
Dretakis et al. [13]	1	Conversion from femoral osteosynthesis to THA	Stryker MAKO®	The robotic assistance promoted the accurate placement of the acetabular component and the precise restoration of the hip joint anatomy and biomechanics.
Yamamura et al. [56]	19	Revision of a primary THA	ROBODOC®	The bone cement was completely removed in all patients, with no instances of perforation or femoral fracture.
Bargar et al. [3]	30	Revision of a primary THA	ROBODOC®	The robotic arm removed the bone cement or, in cementless cases, the fibrous tissue during the reaming and instantly created the cavity for a new cementless implant without exposing the patient to uncertain risks.
Zhang et al. [59]	1	Revision of a primary THA	Stryker MAKO®	The preoperative CT planning and the assistance of the robotic arm allowed for the preservation of the remaining acetabular bone mass.
Zhou et al. [60]	71	Revision of a primary THA	Stryker MAKO®	The RA promoted favourable cup reconstruction in hip revision with acceptable surgical time and blood loss.
Kalavrytinos et al. [19]	1	Conversion from UKA to TKA	Stryker MAKO®	A highly satisfactory result was reported in an 87‐year‐old female affected by the failure of a medial UKA patient one year after the RA revision surgery.
Wallace [54]	4	Conversion from UKA to TKA	Stryker MAKO®	Good post‐operative results with no need for the use of augments thanks to the extreme accuracy of the robotic system.
Yun et al. [57]	34 (*n* = 17 conventional procedure; *n* = 17 RA procedure)	Conversion from UKA to TKA	Stryker MAKO®	No augments were used in the robotic group (vs. 29% in the conventional surgery).
Mancino et al. [33]	51 (*n* = 16 RA revision procedure; *n* = 35 primary robotic TKA)	Conversion from UKA to TKA	Stryker MAKO®	The RA revision procedure was comparable to primary robotic TKA in terms of intraoperative blood loss, post‐operative rehabilitation outcomes, limb alignment, length of hospital stay and post‐operative complications.
Lachance et al. [23]	50	Conversion from UKA to TKA	Stryker MAKO®	Patients who had a primary RA UKA had significantly fewer augments. No difference in terms of range of motion at 1 year, complications or differences in components when comparing robotic and conventional surgery.
Tuecking et al. [53]	40 (*n* = 20 RA revision; *n* = 20 conventional TKA)	Conversion from UKA to TKA	Smith & Nephew NAVIO®	No need for metal augments in any case.Comparable surgery time, alignment accuracy, bone preservation and outcomes when comparing RA revision and conventional TKA.
Raja et al. [37]	3	Conversion from UKA to TKA	Zimmer Biomet ROSA® Knee System	Good outcomes at 6 months concerning radiographic stability, pain and function.
Lachance et al. [24]	3	Conversion from patellofemoral UKA to TKA	Stryker MAKO®	No augments or revision components were necessary. Good early clinical outcomes at KOOS.
Steelman et al. [50]	1	Revision of a primary TKA	Stryker MAKO®	Satisfactory post‐operative component alignment with excellent clinical results 6 months after the revision surgery.
Danoff et al. [11]	6	Revision of a primary TKA	Stryker MAKO®	The robotic technology was used to remove infected bone and make cuts for the new implant, simulating cuts in healthy bone areas if defects were present. Early results were positive, showing the approach's potential to clear infection and restore knee function.
Cochrane et al. [10]	115	Revision of a primary TKA	Smith & Nephew CORI®	Favourable 90‐day outcomes with robotic assistance, with a lower rate of complications, reoperations, and readmissions compared to conventional revision TKA. The use of imageless robotic systems showed lower healthcare costs and radiation exposure compared to image‐based robotic systems.
Ngim et al. [34]	19	Revision of a primary TKA	Stryker MAKO®	The bone cuts necessary for the revision implant were comparable to those performed in a primary case.
MacAskill et al. [27]	25	Revision of a primary TKA	Stryker MAKO®	A statistically significant difference was observed between RA RTKA and primary TKA in terms of posterior condylar offset and tibial slope component positioning. The potential benefit of robotic assistance may include appropriate implant sizing, fundamental for resultant ligamentous tension and a stable revision TKA.
Magruder et al. [28]	41	Conversion from UKA to TKA	Stryker MAKO®	Improved patient‐reported outcomes and low revision and complication rate at 1‐year follow‐up, even in cementless implants (86.5%).
MacAskill et al. [26]	2	Revision of a primary TKA	Stryker MAKO®	RA can improve component alignment and balance, with good post‐operative results. The authors suggest that robotic surgery may reduce the risk of failure in TKA revisions and improve patient satisfaction.

Abbreviations: CT, computed tomography; KOOS, Knee Injury and Osteoarthritis Outcome Score; RA, robotic‐assisted; THA, total hip arthroplasty; UKA, unicompartmental knee arthroplasty.

## DISCUSSION

The results of this scoping review highlight the growing impact and potential of RA systems in the field of revision hip and knee arthroplasty. The most commonly used robotic system was Stryker MAKO®, an image‐based system using CT planning, which most likely enables greater accuracy in preoperative planning, a key factor in revision arthroplasty surgery. Revision arthroplasty, in general, presents increased surgical challenges compared to primary arthroplasty for various reasons, including bone loss, scar tissue and compromised anatomical landmarks. Further complications arise with the highly expected precision for proper positioning and alignment of implants to ensure longevity. Robotic systems, such as MAKO®, ROSA®, ROBODOC®, NAVIO® and CORI®, are proving to be valuable tools in addressing these challenges, by providing increased accuracy in the positioning of the implant, reduced variability in the surgical execution, and bone stock preservation—all critical for the success of revision surgeries. Although the conversion of failed TJA to revision TJA with robotic assistance is currently not approved by the Food and Drug Administration and thus off‐labelled for some of the robotic systems, several studies included in this review demonstrated the benefits of RAprocedures in improving patient outcomes. For example, the work of Zhang et al. [59] and Bargar et al. [3] showcased the role of robotic systems in managing complex acetabular defects, where robotic precision enabled the preservation of remaining bone stock and optimised implant placement. Similarly, studies like those conducted by Lachance et al. [23, 24], Mancino et al. [33], Yun et al. [57], Tuecking et al. [53] and Raja et al. [37] emphasised the role of diverse robotic systems in converting partial knee arthroplasties to total knee arthroplasties with high accuracy, minimising the need for bone augmentation and reducing surgical time. These studies collectively suggest that RA revision arthroplasty may offer superior short‐term results compared to conventional manual techniques.

Another notable advantage of robotic systems is their ability to integrate preoperative imaging with intraoperative execution, allowing surgeons to create patient‐specific surgical plans that account for unique anatomical variations. This integration helps minimise intraoperative adjustments and ensures more predictable results, a crucial factor in revision surgeries where the margin for error is small. For instance, Yamamura et al. demonstrated that robotic systems such as ROBODOC® were able to accurately remove the bone cement in revision procedures without causing additional bone damage and thus facilitated faster recovery with a reduction in the risks of post‐operative complications [56].

However, despite these clear advantages, some significant barriers limit the broader use of RA revision arthroplasty. Among them, the high purchase and maintenance costs of robotic systems remain one of the major deterrents, particularly in resource‐poor settings. Additionally, the steep learning curve associated with these systems requires substantial training and practice, which can delay their integration into regular surgery [31]. This is particularly relevant for revision surgeries, where even minor errors can lead to severe complications such as implant failure or infection. Moreover, while the immediate post‐operative outcomes of RA revision operations are promising, long‐term data on implant survival and overall patient outcomes remain scarce. Current studies, such as those by Yun et al. [57] and Ngim et al. [34], suggest that robotic systems offer similar or slightly better outcomes compared to conventional techniques in the short term. However, questions about the ability of these improvements to lead to extended implant longevity or reduced revision rates are still unanswered. On the other hand, some authors do not report better results in terms of clinical, functional, and radiological outcome, nor increased patient satisfaction after RA arthroplasty [6, 17, 43]. Another significant consideration is the reliance of robotic systems on accurate preoperative imaging, which may be hindered in cases where patients have prior implants that create imaging artefacts. Ngim et al. [34] have discussed how metal artefact reduction systems help mitigate this issue, yet the dependency on advanced imaging technology may limit the applicability of robotic systems in certain revision scenarios. Additionally, the heterogeneity in study designs and outcome measures across the existing literature makes it difficult to draw definitive conclusions about the overall effectiveness of RA revision surgeries compared to conventional methods.

By synthesising the available evidence, this study can present valuable information to delineate the role of RA revision arthroplasty by critically assessing its potential benefits, current constraints, and study gaps. It provides one of the first critical reviews on this underexamined but clinically relevant topic: the systematic search strategy in multiple databases adds validity by ensuring an extensive and representative body of evidence. It might facilitate orthopaedic surgeons' clinical decisions through the analysis of the influence of robotic technology on implant position, bone salvage, and intraoperative precision (key factors in long‐term revision success). Furthermore, this study could contribute to the controversy surrounding the allocation of healthcare funding cost‐benefit analysis, and access to RA technology, and therefore it could be useful for policy‐makers and hospital administrators. While promising early results are available, widespread use of RA revision arthroplasty is currently limited by several factors, including cost, a steep learning curve, and a lack of robust long‐term clinical outcome data. While research in this review has shown improved intraoperative accuracy, we should see whether advances will be reflected in improved long‐term patient results. However, it is only with large, high‐quality trials, including randomised controlled trials with long‐term follow‐up, that the clinical advantage of RA revision over conventional methods can be guaranteed. Overcoming training and economic barriers will be key to facilitating broader integration into routine clinical practice. With continued refinement by the technological progress of RA systems, their use in revision TJA may evolve from an adjunct to standard care, with the potential to improve surgical outcomes and patient satisfaction.

## CONCLUSION

RA systems represent an important evolution in the practice of revision hip and knee arthroplasty by increasing precision during surgery and offering improved short‐term results. The current evidence suggests that robotic systems may reduce surgical variability and lead to improved short‐term outcomes. However, their high cost, steep learning curve, and limited long‐term data restrain widespread adoption. Addressing the economic, training, and logistical challenges associated with robotic systems will be the key to their broader adoption in orthopaedic practice. Though early results are promising, further research is necessary to fully realise the potential of these technologies in revision surgeries.

## AUTHOR CONTRIBUTIONS

**Conceptualisation**: Michele Venosa. **Methodology**: Giandomenico Logroscino and Emilio Romanini. **Formal analysis**: Giuseppe Petralia and Andrea Vespasiani. **Writing—original draft preparation**: Michele Venosa and Gianpiero Cazzato. **Writing—review and editing**: Michele Venosa and Emilio Romanini. **Supervision**: Giacomo Placella and Patrizio Caldora. All authors have read and agreed to the published version of the manuscript.

## CONFLICT OF INTEREST STATEMENT

The authors declare no conflicts of interest.

## ETHICS STATEMENT

The ethics statement is not available.

## Data Availability

The data used to support the findings of this study are available from the corresponding author upon request.
